# Prognostic Impact of Tumor Status, Nodal Status and Tumor Sidedness in Metastatic Colon Cancer

**DOI:** 10.7759/cureus.11444

**Published:** 2020-11-11

**Authors:** Shiva Kumar R Mukkamalla, Ponnandai Somasundar, Bharti Rathore

**Affiliations:** 1 Hematology and Oncology, Ted and Margaret Jorgensen Cancer Center/Presbyterian Healthcare Services, Rio Rancho, USA; 2 Surgical Oncology, Roger Williams Medical Center, Providence, USA; 3 Hematology and Medical Oncology, Roger Williams Medical Center, Providence, USA

**Keywords:** metastatic colon cancer, tumor staging, nodal staging, tumor sidedness, prognosis

## Abstract

Background

Locally advanced primary tumors have been associated with poor overall survival (OS) in non-metastatic colon cancer. However, their impact on metastatic colon cancer (mCC) is not fully defined. The association between primary tumor location and prognosis in mCC is also evolving.

Methods

Using National Cancer Data Base, we identified a cohort of 25,377 patients diagnosed with mCC from 2004-2009. Chi-square test was used for descriptive analyses, while all potential prognostic factors were evaluated using Kaplan-Meier survival estimates and Cox proportional hazards regression modeling.

Results

The five-year OS for the entire study cohort was 12.3%. Factors associated with significant survival impact in multivariate analysis included age, gender, race, comorbidity index, academic level of treating institution, insurance status, income, year of diagnosis, primary tumor site, histologic differentiation, pathologic tumor stage (pT), pathologic nodal stage (pN), and modality of chemotherapy. pT1 lesions demonstrated poor prognosis in stage IV colon cancers, not statistically different when compared to survival outcomes observed in cases with pT4 lesions. Regional nodal involvement demonstrated poor OS in full cohort analysis and subgroup analysis independent of primary tumor location. Both right-sided and transverse colon tumors had similarly worse OS compared to left-sided tumors (right-sided: HR: 1.21, 95% CI: 1.17-1.25; transverse: HR: 1.21, 95% CI: 1.15-1.27).

Conclusions

T1 lesions arising from right-side or transverse colon portend a poor prognosis in mCC, while regional lymph node involvement by itself is an independent poor prognostic factor. Right-sided tumors are associated with poor outcomes than left-sided tumors, suggesting the role of underlying molecular or biologic variants.

## Introduction

Epidemiological reports have always combined colon and rectal cancers for calculation of incidence and mortality rates. According to the most recent cancer statistics data published by the American Cancer Society’s Surveillance and Health Services Research Program, colorectal cancer is the third leading cause of cancer both among men and women in the United States [1]. In 2020, an estimated 147,950 new colorectal cancer cases will be diagnosed in the United States, with an estimated 53,200 deaths. It is now the second leading cause of cancer-related deaths in men and women combined. As of January 2019, there will be an estimated 1.5 million people in the U.S. diagnosed with colorectal cancer [2].

A small proportion of metastatic colon cancer patients with limited metastases confined to liver and/or lungs undergo resection and systemic treatment with curative intent, while the rest are treated with palliative treatment modalities, including systemic therapy with or without palliative resection [3, 4]. Sometimes, radiofrequency ablation or external beam radiation is also utilized to address oligometastatic disease in the liver. Several small registries and single institution-based studies have looked into the impact of various clinic-pathologic features on disease prognosis in stage IV colon cancer [5-8]. Few of these studies also evaluated the prognostic impact of primary tumor resection.

The current study was undertaken to assess the prognostic impact of various patient-, tumor- and treatment-specific factors in stage IV colon cancer patients who underwent palliative resection of the primary tumor, utilizing a large population-based cohort extracted from the National Cancer Data Base (NCDB). This study cohort included patients treated with palliative intent only while excluding those who underwent metastasectomy. Our study objective was to identify the prognostic impact of tumor status (T), nodal involvement (N), and location in stage IV colon cancers, managed with palliative intent.

## Materials and methods

Data source

The NCDB is a joint quality improvement initiative of the American College of Surgeons’ Commission on Cancer and the American Cancer Society. It contains 34 million patient records and represents approximately 70% of all newly diagnosed cancer cases in the United States [9]. Data was provided through Participant User File (PUF) by the NCDB after a thorough review of the application. The PUF included patients diagnosed with colon cancer from 2004 to 2014.

Study population

Patients diagnosed with stage IV colon adenocarcinoma from 2004 to 2009, who underwent palliative resection of the primary tumor, were identified from the NCDB. Patients undergoing metastasectomy were excluded from the study population, and those cases with missing or unknown data were also excluded from the analysis. Using the American Joint Committee on Cancer’s (AJCC) Collaborative Stage Data Collection System (CCS), we excluded patients with appendiceal and overlapping adenocarcinomas. A final cohort of 25,377 patients was identified for analysis. Only cases with pathologic confirmation were included in the final cohort. As a cut-off point, the year 2009 was chosen to ensure a minimum follow-up of five years for all patients included in the analysis.

Outcomes and variables

Five-year overall survival was chosen as the primary endpoint for the study. Censoring was performed at the last recorded follow-up of 60 months. The variables of interest were age, gender, race, Charlson/Deyo comorbidity score, academic level of treating institution, geographic region, rural-urban continuum, insurance status, median family income, year of diagnosis, location of the primary tumor, differentiation of tumor, histologic subtypes of adenocarcinoma, pathologic AJCC tumor status (T), pathologic AJCC nodal status (N), chemotherapy, surgical resection of primary tumor and receipt of palliative external beam radiation.

Age was recoded into ordinal groups of 18-64 years, 65-74 years, and over 75 years. Charlson/Deyo comorbidity index was used to stratify the study population into three different relative risk categories: (i) patients with score zero, (ii) those with a score of one and, (iii) patients with a score of two or more, with zero indicating no comorbidities at baseline. Tumor locations were divided into three categories: left-sided, right-sided, or transverse colon tumors. Median family income was analyzed as four quartiles representing income levels ranging from less than $38,000 to more than $63,000. Tumors were graded into well-differentiated, moderately differentiated, and undifferentiated tumors. Based on the histology of the tumor, cases were classified into mucinous or non-mucinous adenocarcinomas. A tumor (T), nodal (N), and metastasis (M) staging was based on AJCC, 6th edition.

Statistical analysis

Statistical analysis was performed using the Statistical Analysis Software (SAS, version 9.4; SAS Institute, Cary, NC). Kaplan-Meier survival analysis was utilized to generate adjusted survival curves and compared using the log-rank test. We conducted this part of the analysis using the SAS procedure PROC GPLOT. Cox proportional hazards regression model was used to perform multivariate analysis to assess the influence of the above-mentioned variables by generating hazards ratios (HR) and 95% confidence intervals (CI), with an HR less than 1.0 indicating survival benefit. Multivariate regression analysis was completed using SAS procedure PROC PHREG. Differences were considered statistically significant when the p-value was <0.05.

## Results

Patient characteristics

A cohort of 25,377 stage IV colon cancer patients diagnosed from 2004 to 2009, containing information on all the variables of interest, was identified from the NCDB. Patient demographics and clinicopathologic features of the entire study cohort are demonstrated in Table 1. The five-year overall survival rate for this cohort was 12.3%, which is similar to the national average for stage IV colon cancer [10]. Approximately, 50% of the study population was less than 65 years old and Caucasians had majority representation among various ethnic groups (77%). Most of the patients had no baseline comorbidities (73.7%) and almost three-fourths of the study population was treated at non-academic centers. With regards to the rural-urban continuum, almost 85% of patients were from metropolitan areas and 95.6% of the entire study cohort had some kind of health insurance. Right-sided tumors constituted almost half of the patient population (49.6%) while mucinous adenocarcinoma represented only 11.5% of all tumors. T1 tumors formed a very small minority (0.6%) of the entire study cohort, which was otherwise predominantly represented by T3 tumors (62%). More than half of them had N2 disease. Approximately 60% of the study population underwent subtotal colectomy. Palliative external beam radiation was administered in only 2.7% of patients and more than half of the study cohort received multi-agent chemotherapy.

**Table 1:** Patient characteristics AJCC -  American Joint Committee on Cancer; pT - pathologic tumor stage; pN - pathologic nodal stage

Characteristics	No. of patients (%)
Age	
18-64 years	12,636 (49.8)
65-74 years	6073 (23.9)
≥75 years	6668 (26.3)
Gender	
Male	12,406 (48.9)
Female	12,971 (51.1)
Race	
White	19,539 (77)
Black	3798 (15)
Hispanic	1160 (4.6)
Others	880 (3.4)
Charlson/Deyo comorbidity score	
0	18,704 (73.7)
1	5128 (60.2)
≥2	1545 (6.1)
Institution	
Academic	6575 (26.9)
Non-academic	17,851 (73.1)
Region	
East North Central	4514 (18.5)
East South Central	1783 (7.3)
Middle Atlantic	3281 (13.4)
Mountain	1035 (4.2)
New England	1295 (5.3)
Pacific	2776 (11.4)
South Atlantic	5932 (24.3)
West North Central	1802 (7.4)
West South Central	2008 (8.2)
Location	
Metro	20,358 (84.3)
Urban	3372 (14)
Rural	411 (1.7)
Insurance	
Insured	24,268 (95.6)
Uninsured	1109 (4.4)
Median family income	
less than $38,000	4923 (20)
$38,000-$47,999	6035 (24.6)
$48,000-$62,999	6307 (25.7)
≥$63,000	7297 (29.7)
Year of diagnosis	
2004	4344 (17.1)
2005	4300 (16.9)
2006	4287 (16.9)
2007	4155 (16.4)
2008	4280 (16.9)
2009	4011 (15.8)
Site of primary tumor	
Left side	10,574 (41.7)
Right side	12,574 (49.6)
Transverse colon	2229 (8.8)
Tumor differentiation	
Well differentiated	1293 (5.1)
Moderately differentiated	16,107 (63.5)
Poor/Undifferentiated	7977 (5.1)
Histology	
Non-mucinous adenocarcinoma	22,449 (88.5)
Mucinous adenocarcinoma	2928 (11.5)
AJCC pT	
T1	145 (0.6)
T2	696 (2.7)
T3	15,732 (62)
T4	8804 (34.7)
AJCC pN	
N0	4101 (16.2)
N1	7895 (31.1)
N2	13,381 (52.7)
Chemotherapy	
None	8348 (32.9)
Single-agent	1817 (7.2)
Multi-agent	13,506 (53.2)
Type not specified	1706 (6.7)
Colectomy	
Partial	8860 (34.9)
Subtotal	15,242 (60.1)
Total	1275 (5)
Palliative radiation	
Received	678 (2.7)
Not received	24,699 (97.3)
5-year overall survival	
Alive	3110 (12.3)
Dead	22,267 (87.7)

Independent predictors of overall survival

**Figure 1:**
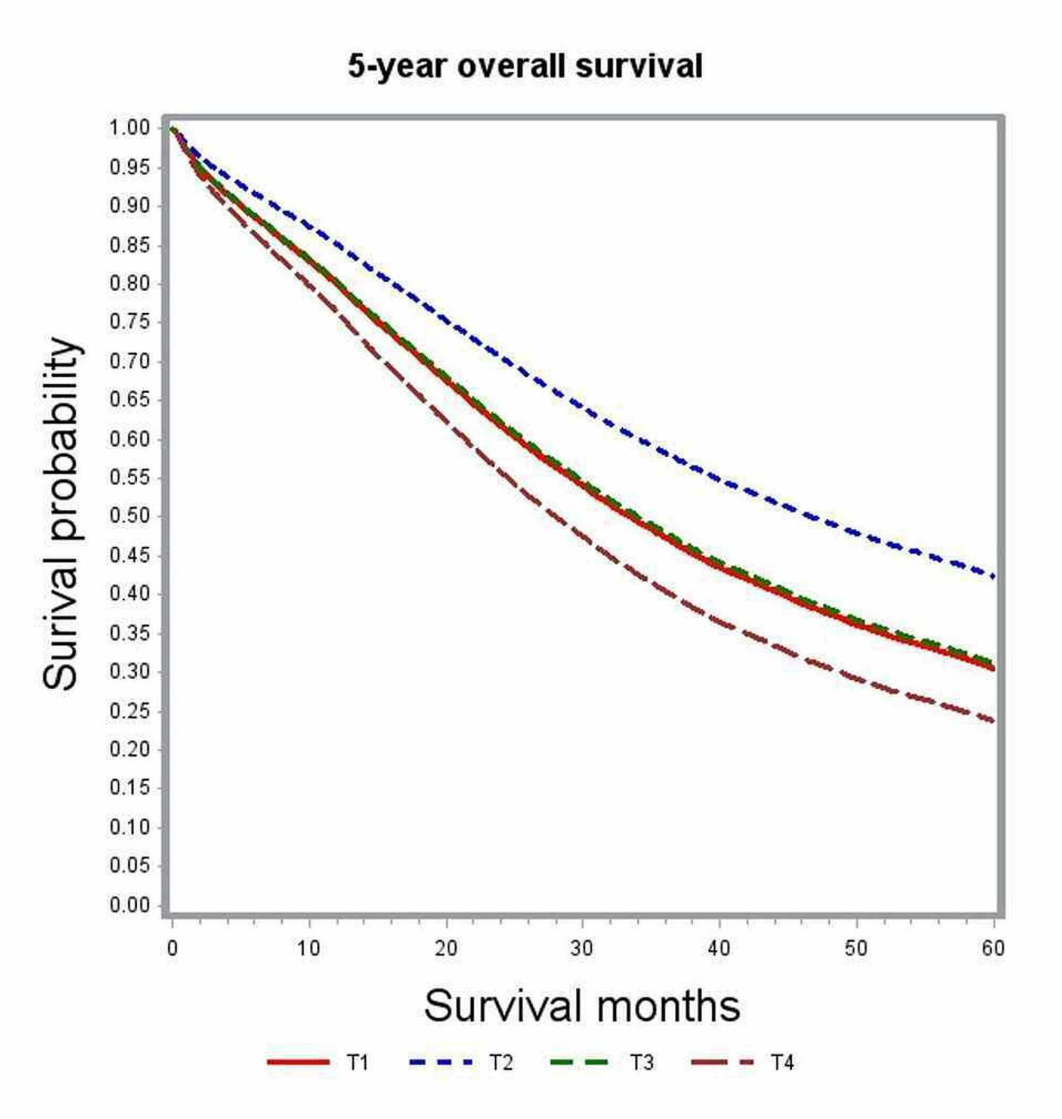
Adjusted survival curves for the entire study cohort stratified by tumor status (T)

**Figure 2:**
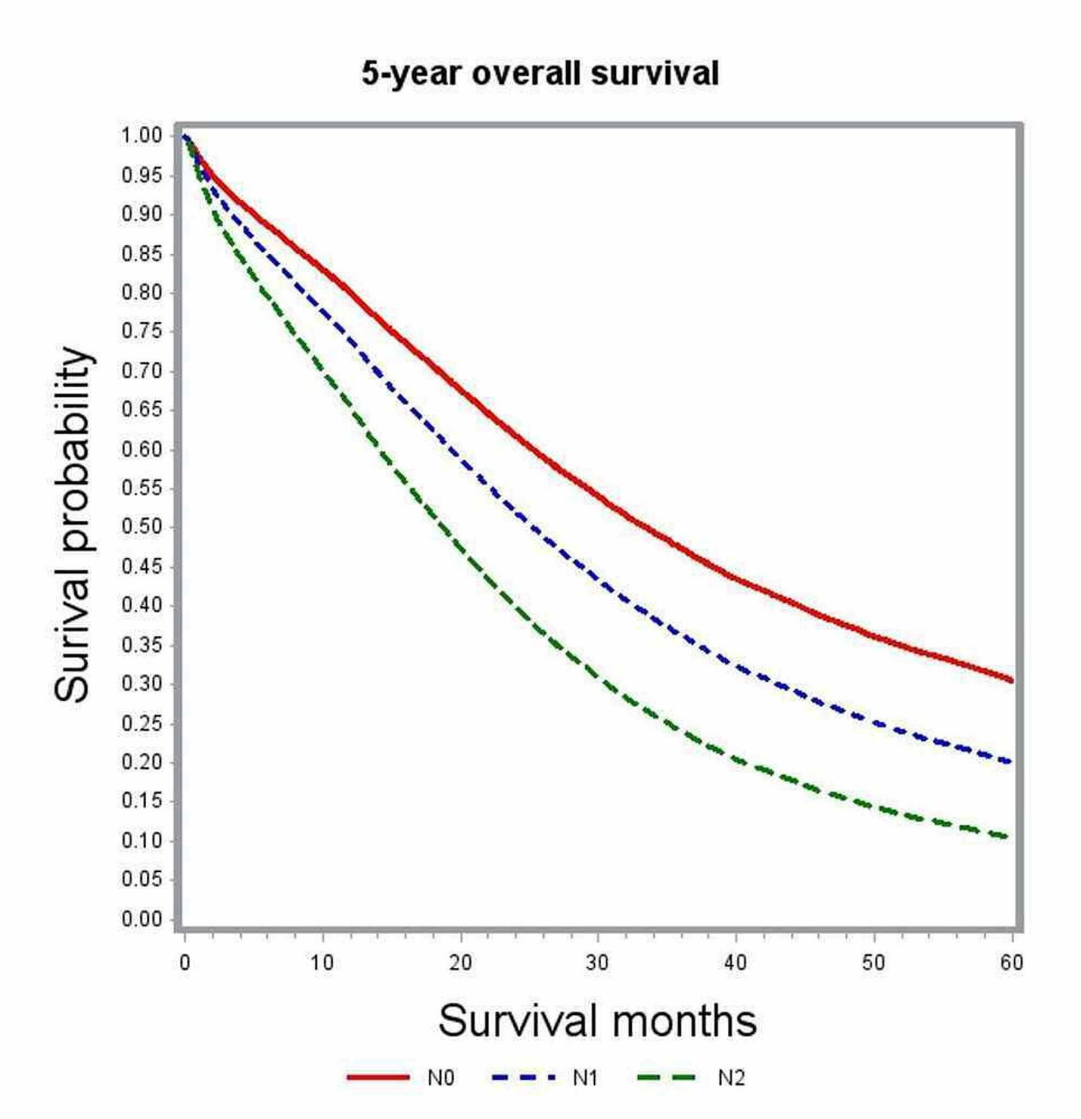
Adjusted survival curves for the entire study cohort stratified by nodal status (N)

**Figure 3:**
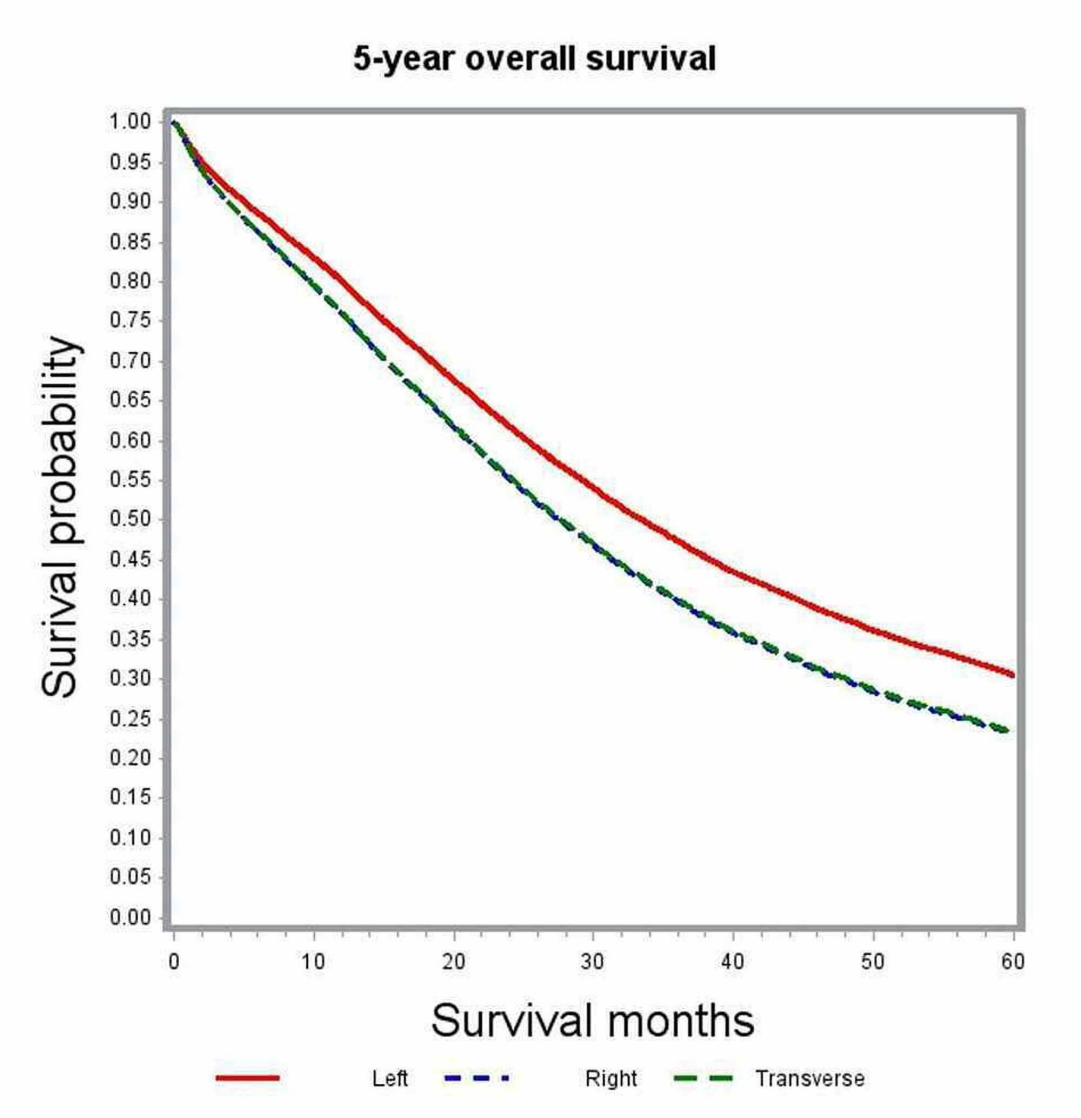
Adjusted survival curves for the entire study cohort stratified by site of the primary tumor

In multivariate analysis, variables associated with significant impact on overall survival included age, gender, race, comorbidity index, academic level of treating institution, certain geographic regions, insurance status, median family income, year of diagnosis, primary tumor site, histologic grade, pathologic tumor stage (pT), pathologic nodal stage (pN) and modality of chemotherapy (Table 2). The unadjusted median survival for patients with T1, T2, and T3 lesions was 19.5 months, 25.6 months, and 21.2 months, respectively. After adjusting for various patient, tumor, and treatment factors, the probability of death was not significantly different between patients with T1 and T4 lesions (HR: 0.83; 95% CI: 0.69-1.00). Figure 1 shows the adjusted survival curves for various T groups. Patients with increasing nodal involvement had decreasing median survival estimates. Patients with no nodal involvement had better outcomes compared to N2 nodal status (HR: 0.55; 95% CI: 0.53-0.58), as depicted in the adjusted survival curves shown in Figure 2. Patients with right-sided tumors had poorer overall survival compared to left-sided tumors (HR: 1.21; 95% CI: 1.17-1.25). Figure 3 demonstrates the adjusted relation between tumor sidedness and improved survival. These survival curves reiterate the lack of survival difference between right-sided and transverse colon tumors, which is also demonstrated by similar HR (right-side: HR: 1.21; 95% CI 1.17-1.25; transverse: HR: 1.21; 95% CI: 1.15-1.27). Chemotherapy use and surgical resection were independently associated with improved survival. Multi-agent chemotherapy administration was associated with improved survival compared to single-agent chemotherapy (HR: 0.85; 95% CI: 0.80-0.9), while not receiving chemotherapy portended poor survival outcomes compared to receiving any modality of chemotherapy.

**Table 2:** A five-year overall survival analysis using multivariate Cox proportional hazards regression model AJCC -  American Joint Committee on Cancer; pT - pathologic tumor stage; pN - pathologic nodal stage

Variable	Median survival (months)	Hazards ratio	95% confidence interval	p
Age				
≥75 years	8.3	Ref=1		
18-64 years	23.5	0.62	0.6-0.64	<0.0001
65-74 years	17.1	0.76	0.73-0.79	<0.0001
Gender				
Male	16.8	Ref=1		
Female	18.6	0.96	0.94-0.99	0.0121
Race				
Black	17.5	Ref=1		
White	17.6	0.93	0.9-0.97	0.0012
Hispanic	20.1	0.83	0.77-0.9	<0.0001
Others	19.8	0.88	0.81-0.96	0.0055
Charlson/Deyo comorbidity score				
≥2	9.6	Ref=1		
0	19.1	0.76	0.72-0.81	<0.0001
1	15	0.84	0.79-0.9	<0.0001
Institution				
Non-academic	16.2	Ref=1		
Academic	21	0.88	0.85-0.91	<0.0001
Region				
Pacific	18.5	Ref=1		
East North Central	16.8	1.09	1.03-1.15	0.0025
East South Central	16.4	1.02	0.95-1.09	0.6431
Middle Atlantic	18.3	1.06	1.01-1.13	0.031
Mountain	16.3	1.02	0.94-1.1	0.6397
New England	18	1.01	0.93-1.09	0.8387
South Atlantic	17.6	1.01	0.96-1.07	0.5936
West North Central	17.2	1.09	1.02-1.17	0.0101
West South Central	17	1	0.94-1.07	0.9996
Location				
Metro	17.9	Ref=1		
Urban	17.3	0.98	0.94-1.02	0.3728
Rural	15.3	0.97	0.88-1.08	0.974
Insurance				
Uninsured	17.6	Ref=1		
Insured	19.2	0.85	0.8-0.92	<0.0001
Median family income				
less than $38,000	16.1	Ref=1		
$38,000-$47,999	17.2	0.98	0.94-1.02	0.2429
$48,000-$62,999	18	0.93	0.9-0.98	0.0019
≥$63,000	19.4	0.88	0.84-0.92	<0.0001
Year of diagnosis				
2004	15.7	Ref=1		
2005	16.8	0.96	0.91-1.01	0.0805
2006	17.6	0.93	0.87-0.97	0.0021
2007	18.5	0.94	0.89-0.98	0.0081
2008	18.6	0.94	0.9-0.99	0.0203
2009	19.4	0.91	0.87-0.96	0.0001
Site of primary tumor				
Left side	23	Ref=1		
Right side	14.3	1.21	1.17-1.25	<0.0001
Transverse colon	15.5	1.21	1.15-1.27	<0.0001
Tumor differentiation				
Well differentiated	23.1	Ref=1		
Moderately differentiated	21	1.07	1.01-1.14	0.0317
Poor/Undifferentiated	11.8	1.46	1.36-1.56	<0.0001
Histology				
Mucinous adenocarcinoma	15.2	Ref=1		
Non-mucinous adenocarcinoma	18.1	0.98	0.94-1.02	0.3033
AJCC pT				
T4	14.3	Ref=1		
T1	19.5	0.83	0.69-1.00	0.05
T2	25.6	0.63	0.58-0.69	<0.0001
T3	21.2	0.84	0.81-0.86	<0.0001
AJCC pN				
N2	14.4	Ref=1		
N0	25.5	0.55	0.53-0.58	<0.0001
N1	21.1	0.74	0.72-0.76	<0.0001
Chemotherapy				
Single-agent	17.5	Ref=1		
Multi-agent	24.8	0.85	0.80-0.9	<0.0001
Type not documented	23.9	0.88	0.82-0.95	0.0007
None	5	1.99	1.88-2.1	<0.0001
Colectomy				
Partial	20.5	Ref=1		
Subtotal	16.1	0.98	0.95-1.02	0.2853
Total	19.8	0.95	0.89-1.02	0.1415
Palliative radiation				
Received	16.7	Ref=1		
None received	17.7	0.95	0.87-1.04	0.2324

Prognostic impact of T and N by tumor location

**Figure 4:**
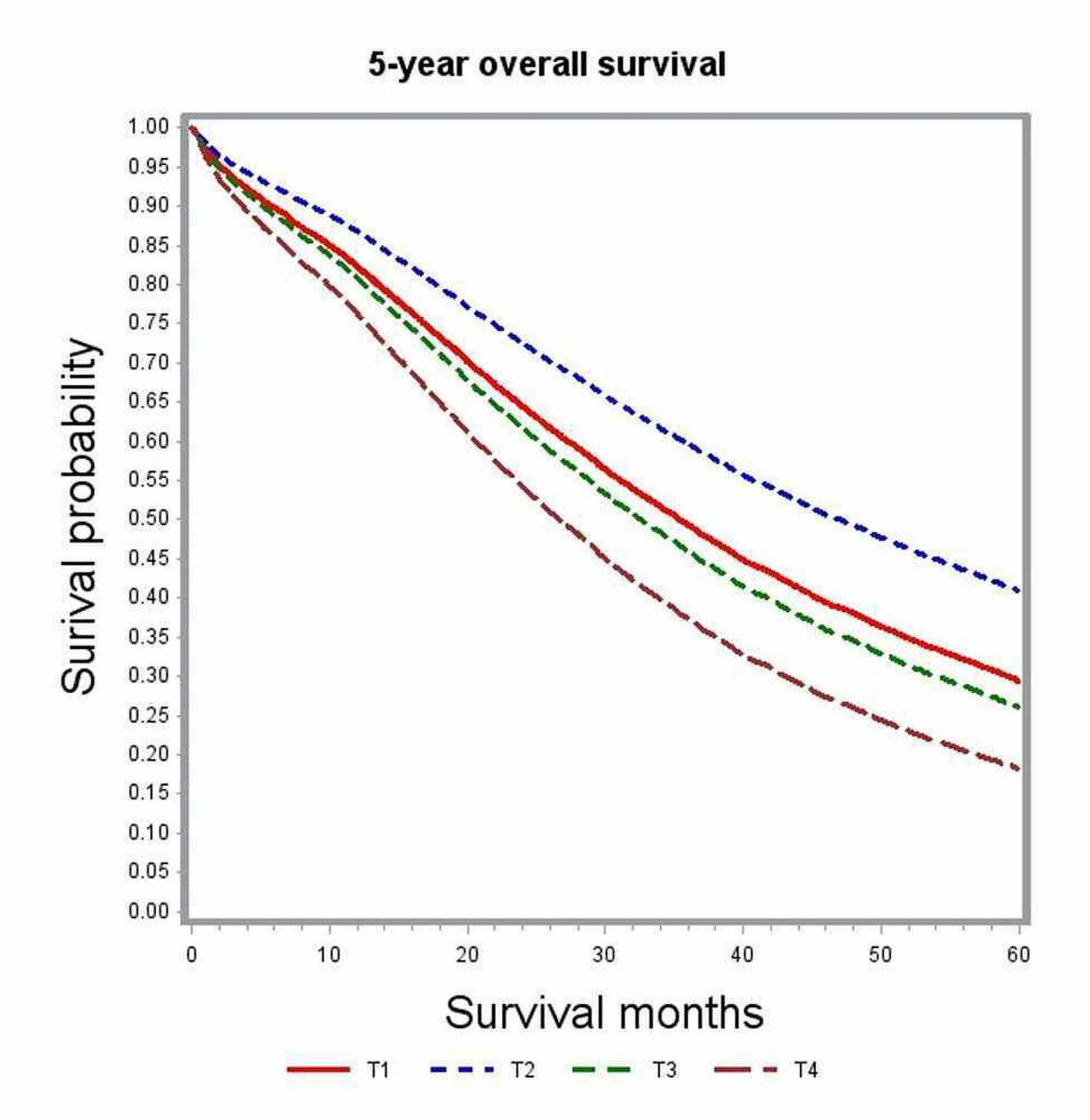
Adjusted survival curves for left-sided tumors stratified by tumor status (T)

**Figure 5:**
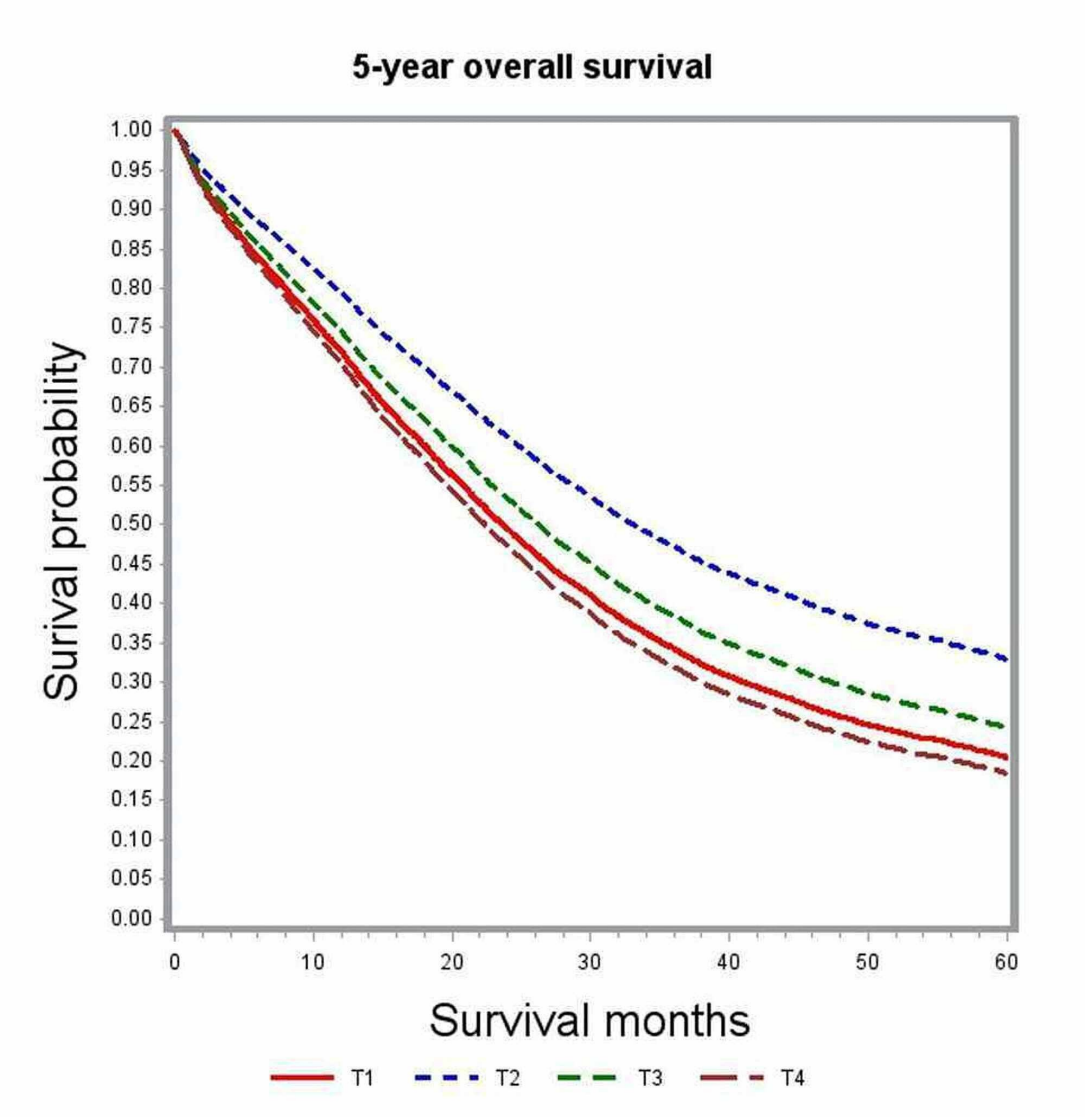
Adjusted survival curves for right-sided tumors stratified by tumor status (T)

**Figure 6:**
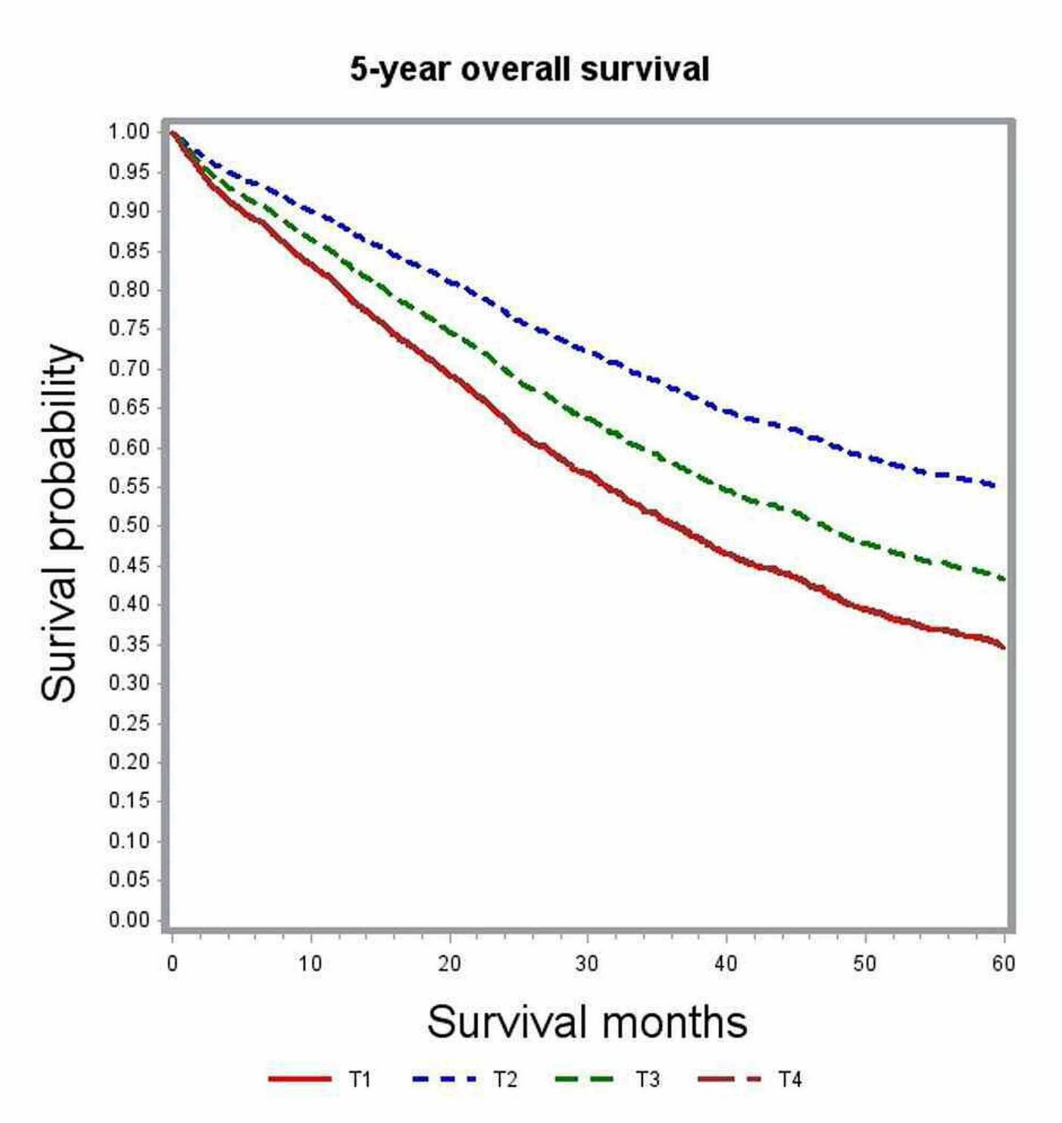
Adjusted survival curves for transverse colon tumors stratified by tumor status (T)

**Figure 7:**
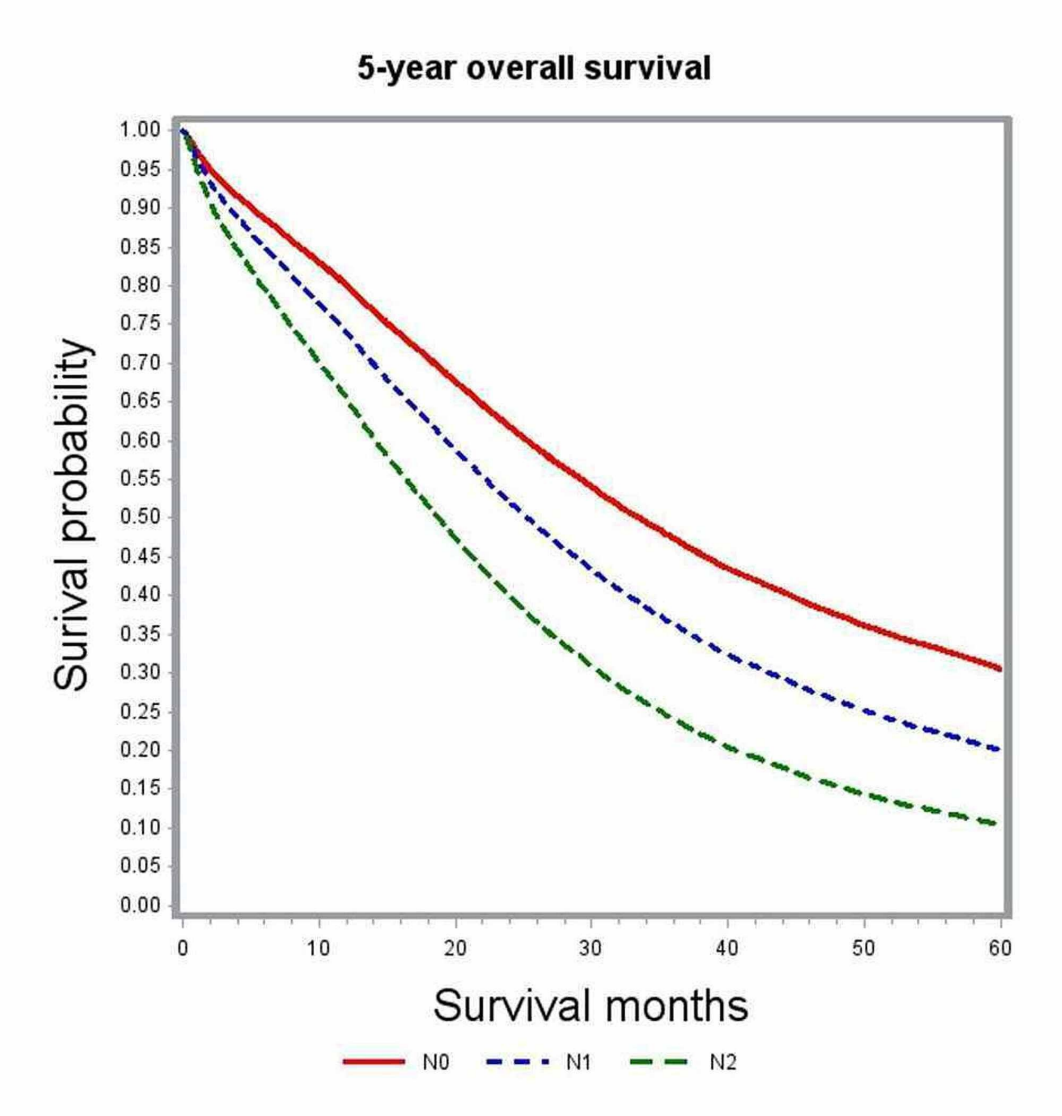
Adjusted survival curves for left-sided tumors stratified by nodal status (N)

**Figure 8:**
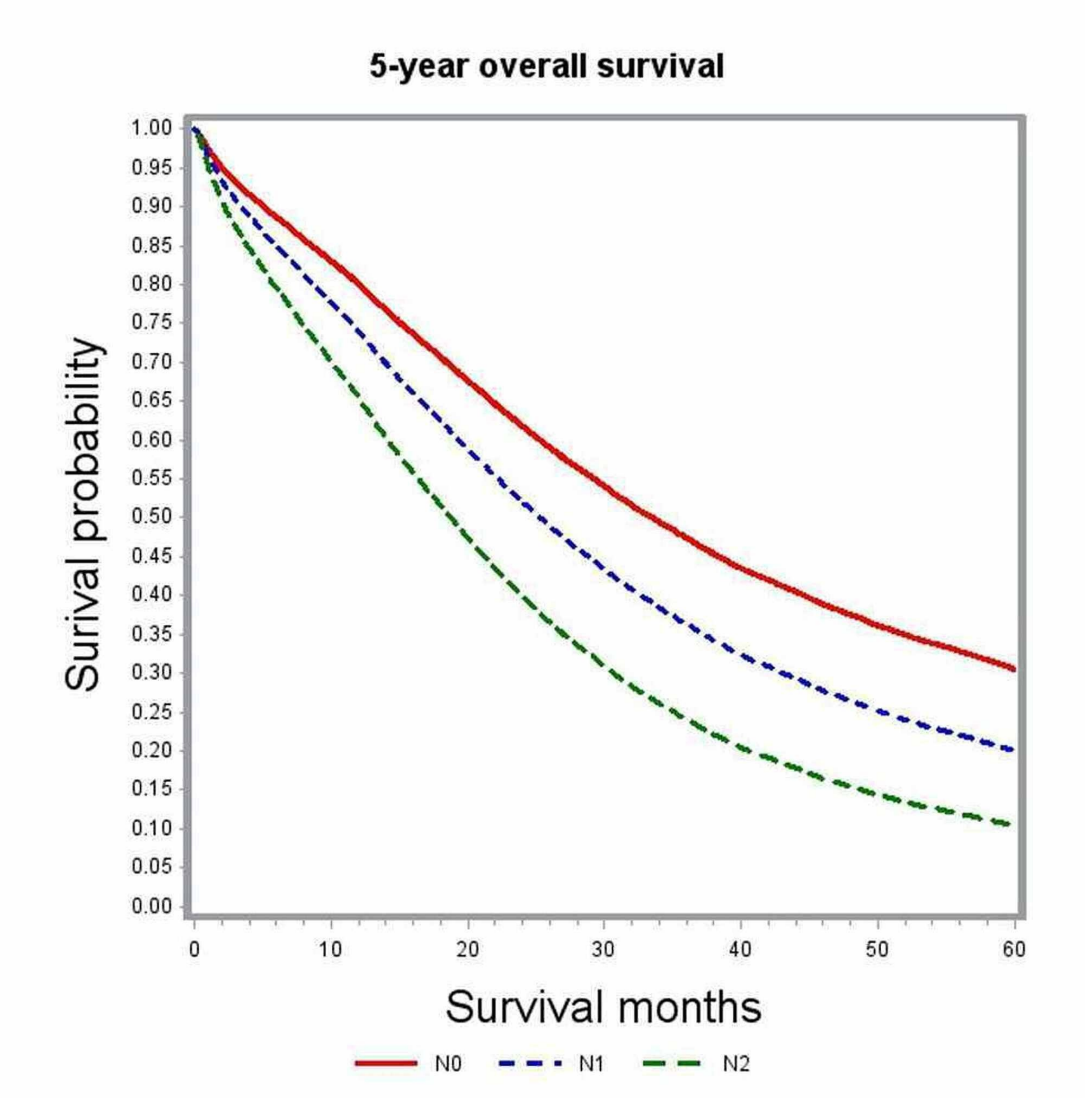
Adjusted survival curves for right-sided tumors stratified by nodal status (N)

**Figure 9:**
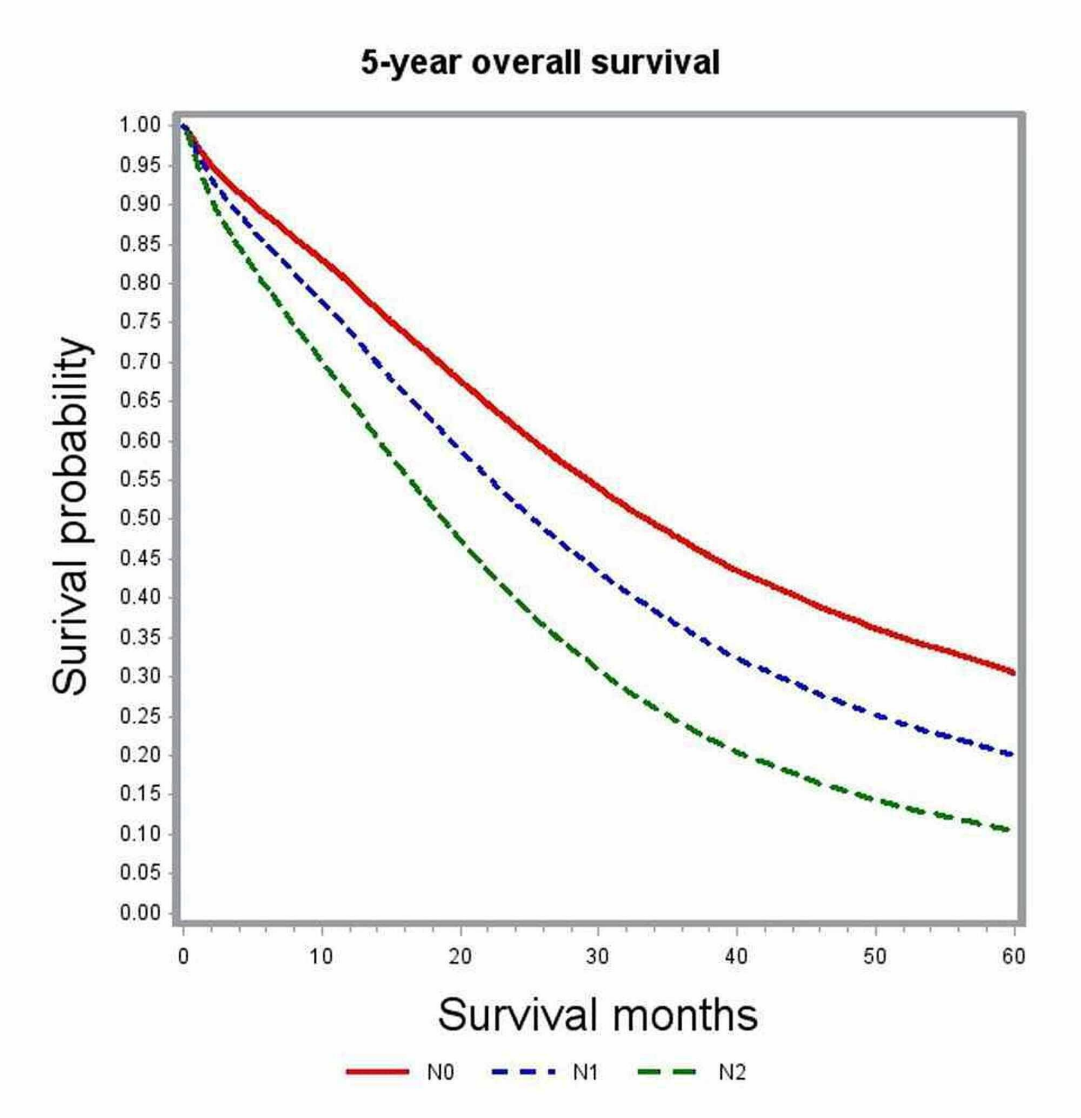
Adjusted survival curves for transverse colon tumors stratified by nodal status (N)

Subgroup analysis by tumor location was performed to further delineate into the prognostic differences seen among various T and N stages (Table 3). After adjusting for covariates, T1 and T4 tumors did not show any significant difference in survival outcomes for right-sided (HR; 0.99; 95% CI: 0.76-1.30) and transverse colon tumors (HR: 1.08; 95% CI: 0.4-2.95). Among left-sided tumors, T1 lesions had better survival outcomes compared to T4 lesions (HR: 0.71; 95% CI: 0.54-0.93) but performed poorly compared to T2 lesions (Figures 4-6). Increasing nodal involvement continued to portend a poor prognosis, irrespective of tumor location (Figures 7-9).

**Table 3:** Multivariate subgroup analysis of five-year overall survival, according to tumor sidedness, primary tumor status (T) and nodal status (N) Variables analyzed as part of multivariate analysis include age, gender, race, Charlson/Deyo comorbidity score, academic level of treating institution, region, location, insurance status, median family income, year of diagnosis, histology, tumor differentiation, chemotherapy, palliative radiation and surgery.

	fFve-year survival, %	HR	95% CI	p
Left-sided (n=10,663)				
Tumor status				
T4	11.7	Ref=1		
T1	18.7	0.71	0.54-0.93	0.0428
T2	30.7	0.56	0.47-0.65	<0.0001
T3	17.3	0.81	0.77-0.85	<0.0001
Nodal status				
N2	12	Ref=1		
N0	23.5	0.62	0.58-0.66	<0.0001
N1	16.7	0.81	0.77-0.85	<0.0001
Right-sided (n=12,664)				
Tumor status				
T4	6.5	Ref=1		
T1	14.1	0.99	0.76-1.30	0.6971
T2	21.3	0.69	0.61-0.78	<0.0001
T3	10.4	0.86	0.83-0.90	<0.0001
Nodal status				
N2	5.5	Ref=1		
N0	19.2	0.51	0.48-0.54	<0.0001
N1	12.7	0.7	0.67-0.73	<0.0001
Transverse colon (n=2259)				
Tumor status				
T4	9.2	Ref=1		
T1	16.7	1.08	0.4-2.95	0.8731
T2	18.9	0.62	0.42-0.9	0.0112
T3	13.1	0.84	0.76-0.92	0.0004
Nodal status				
N2	7.9	Ref=1		
N0	20.8	0.52	0.45-0.59	<0.0001
N1	12.4	0.76	0.69-0.85	<0.0001

## Discussion

We evaluated metastatic colon cancer patients to identify factors that have a prognostic impact on overall survival. This is the first study using NCDB data to systematically evaluate the prognostic implications of various patient-, disease- and treatment-specific factors on overall survival in stage IV colon cancer. Previous studies have demonstrated the survival benefit of palliative tumor resection in stage IV colorectal cancers [11-13]. Therefore, only patients undergoing primary tumor resection were included in our study cohort.

Multivariate analysis of the full cohort demonstrated patients with pT1 lesions had survival outcomes that were not different from those with pT4 lesions (HR: 0.83; 95% CI: 0.69-1.00). However, earlier studies reported that higher T stage was independently associated with poor survival compared to lower T staging [6, 8]. We then performed additional sub-group analysis based on primary tumor location. Multivariate analysis of subgroups based on tumor location showed pT4 and pT1 lesions to have no significant difference in survival outcome both in the right-sided and transverse colon cancers (right-sided: HR: 0.71; 95% CI: 0.54-0.93; transverse: HR: 1.08; 95% CI: 0.4-2.95). Among cancers originating from the left colon, pT1 was associated with better overall survival compared to pT3 and pT4 lesions but performed poorly compared to pT2 lesions (Table 3). In the full cohort as well as subgroup analysis, pT2 was independently associated with the best survival outcomes. We further looked into the distribution of cases according to nodal status stratified by tumor stage (Table 4) but could not identify any significant association between pT and pN to explain this intriguing observation. We could hypothesize that T1 lesions may represent a biologically distinct tumor that is more aggressive with unique defects in various molecular and biologic pathways that were not accounted for in this retrospective study.

**Table 4:** Nodal involvement stratified by tumor stage (chi-square test, p <0.0001) AJCC -  American Joint Committee on Cancer; pT - pathologic tumor stage; pN - pathologic nodal stage

AJCC pT	AJCC pN, n (%)			Total, N
	N0	N1	N2	
T1	54 (37.2)	66 (45.5)	25 (17.2)	145
T2	241 (34.6)	269 (38.7)	186 (26.7)	696
T3	2526 (16.1)	5175 (32.9)	8031 (51)	15,732
T4	1280 (14.5)	2385 (27.1)	5139 (58.4)	8804
Total, N	4101	7895	13,381	25,377

Nodal involvement was found to be an independent predictor of poor survival. Multivariate survival analysis showed that patients with N1 or N2 disease performed poorly compared to those with no nodal involvement (N0: HR: 0.55, 95% CI: 0.53-0.58). Median survival in patients with N0 disease was 25.5 months as compared to 21.1 months with N1 and 14.4 months in N2 nodal involvement. This difference remained significant independent of primary tumor location, as confirmed in the subgroup analysis (Table 3). These results are consistent with the findings reported in previous retrospective studies [8, 14-20]. The proportion of patients with N0 disease in our study was 16%, which is similar to the frequency of 15-20% that was reported in these earlier studies. Since regional lymph node metastasis is considered an early sign of distant metastasis, the presence of 15-20% of node-negative cases in stage IV colon cancers remains quite inexplicable. There is paucity in the literature about various biological mediators related to lymphatic spread in colorectal cancers. Kawada et al. demonstrated the chemokine receptor CXCR-3 positive colon cancers to have poor survival than those without CXCR-3 [21]. It was postulated that patients lacking biologic mediators, like CXCR-3 related to lymphatic spread, may have tumor with less aggressive traits and carry a favorable prognosis.

Location of primary tumor also proved to be an independent factor of overall survival in our study, with right-sided colon cancer showing significantly worse survival compared to left-sided colon cancer (HR: 1.21; 95% CI: 1.17-1.25). Previous studies looking into primary tumor location and outcomes have reported discordant results [22]. However, these studies included a heterogeneous population with different stages receiving non-uniform treatments. According to Surveillance, Epidemiology and End Results (SEER) database analysis by Meguid et al., the prognosis of right-sided tumors was not different from that of left-sided tumors in stage I cancer, while it was shown to be better in stage II cancers and worse in stage III and IV disease [22]. A more recent SEER database analyses by Schrag et al. reported right-sided tumors to have inferior survival in patients with stage III and IV colon cancer after adjusting for various clinical and demographic characteristics [23].

In the Cancer and Leukemia Group B/South West Oncology Group (CALGB/SWOG) 80405 analysis by Venook et al., tumor sidedness was shown to have both prognostic and predictive value in metastatic colon cancer [24]. In this analysis, patients with left-sided Kirsten rat sarcoma viral oncogene homolog (KRAS) wild-type disease were found to have better overall survival and progression-free survival than those with KRAS wild-type right-sided cancer. In addition, survival outcomes favored treatment with bevacizumab than cetuximab in patients with right-sided primary tumors. A retrospective analysis of rat sarcoma viral oncogene (RAS) wild-type metastatic colorectal cancer patients from Cetuximab Combined With Irinotecan in First-Line Therapy for Metastatic Colorectal Cancer (CRYSTAL) and FOLFIRI Plus Cetuximab Versus FOLFIRI Plus Bevacizumab as First-Line Treatment of KRAS Wild-Type Metastatic Colorectal Cancer (FIRE-3) reported left-sided cancers to have a better prognosis in terms of survival and response rates, irrespective of treatment [25].

Right-sided colon cancers tend to be diagnosed much later than left-sided colon cancers. This clinical observation highlights the tendency for right-sided colon cancers do not produce symptoms until they are relatively advanced with larger tumor size, invasion to adjacent organs, the involvement of regional lymph nodes, and multiple organ metastases. This difference in presentation time-line has been argued to result in a “lead-time” bias for left-sided tumors that may make survival appear longer. All of these prior studies that looked into left-sided vs. right-sided tumors alone when assessing for the difference in survival. However, it is known that during embryonic development, the right side of the colon and proximal two-thirds of the transverse colon develops from the midgut while the left side of the colon and distal one-third of the transverse colon originate from the hindgut. Our study analyzed the site of the primary tumor as originating from either left, right, or transverse colon. By doing this, we were able to show the close similarity between the survival outcomes of right-sided and transverse colon tumors (right-sided: HR: 1.21; 95% CI: 1.17-1.25; transverse: HR: 1.21; 95% CI: 1.15-1.27).

Right-sided colon cancers are characterized by a high frequency of microsatellite instability (MSI) and BRAF mutation [26-29]. BRAF mutation has been shown to carry a poor prognosis. MSI has been shown to impart favorable prognosis in curatively resected stage II colon cancers but is associated with poorer survival in patients with stage IV colorectal cancers. Guinney et al. looked into various gene expression arrays of colorectal cancers and came up with four well-defined consensus molecular subtypes (CMS), which reflect the way colorectal cancer behaves biologically [30]. CMS1 and CMS3 are associated with poor prognosis, and these two subtypes were more likely to be present in the right side of the colon.

There are some potential limitations to our current study. First, information on MSI, BRAF mutational status, or any other molecular correlates was lacking. Although tumor sidedness could be considered a surrogate for these high-risk biological subtypes, this could not be validated in the current study cohort. Second, this is a non-randomized, retrospective analysis that could carry an inherent selection bias. However, we tried to minimize heterogeneity by including only stage IV colon cancer patients who underwent palliative resection and excluding cases undergoing curative metastasectomy. Finally, NCDB does not collect information about disease-specific mortality, and the overall survival outcomes discussed in the current study might have been influenced by secondary competing mortality.

Despite these limitations, the current analysis is the first and largest population-based analysis of metastatic colon cancer patients, utilizing the NCDB.

## Conclusions

Using a large national database, we demonstrated pT1 lesions to carry a poor prognosis in stage IV colon cancers, not statistically different when compared to survival outcomes observed in cases with pT4 lesions. This observation was validated in the right-sided and transverse colon only subgroups, highlighting the impact of various underlying distinct biological subtypes. Regional lymph node involvement was associated with poorer overall survival in metastatic colon cancer patients undergoing palliative resection of the primary tumor. Most importantly, right-sided tumors demonstrated worse prognosis than left-sided tumors. This further strengthens the idea of tumor-sidedness being a surrogate marker for underlying high-risk biological or molecular subtypes, which needs additional prospective validation in clinical trials.
